# Provision of care to hospitalized pediatric burn patients: a qualitative study among nurses at Muhimbili National Hospital, Dar es Salaam, Tanzania

**DOI:** 10.1186/s12912-019-0335-1

**Published:** 2019-03-12

**Authors:** Nyakanda P. Marwa, Edith A. M. Tarimo

**Affiliations:** 1 Muhimbili National Hospital, P O Box 65000, Dar es Salaam, Tanzania; 2 Muhimbili University of Health and Allied Sciences, School of Nursing, P.O Box 65004, Dar es Salaam, Tanzania

**Keywords:** Burn, Injury, Care, Pediatric, Nurses, Dar Es Salaam, Tanzania

## Abstract

**Background:**

Burn injury is a significant problem in low and middle-income countries. Moreover, across regions children are more affected by burn injury than adults. The outcome of burn injury is greatly influenced by the quality of care patients receive. This care includes meeting nutritional needs, availability of resources such as dressing supplies, and skills among health care providers. This study describes factors that influence provision of nursing care to the hospitalized pediatric patients with burn injuries at Muhimbili National Hospital, Dar es Salaam, Tanzania.

**Methods:**

A descriptive qualitative study was conducted among registered nurses working in the Pediatric Burn Unit. Purposeful sampling was used to recruit the participants in the study. Five in-depth interviews were done and content analysis approach was used.

**Results:**

The nurses in the study described how they provided nursing care to pediatric patients with burn injuries. They described the use of closed method wound dressing, as an essential skill that accelerated wound healing, decreased the risk of wound contamination, and the incidence of contractures. The nurses felt gratified when they saw patients who had sustained severe burn injury recover well and be discharged home. They appreciated the influence of teamwork in burn patients’ recovery. However, the interviews revealed systematic deficiencies that hindered provision of quality care to patients with burn injuries. The flaws included: inadequate staffing resulting in increased workload among the nurses; a lack of standard skills in burn care among nurses; lack of access to water, which is the mainstay of infection prevention control, and lack of specimen collection equipment.

**Conclusions:**

Findings in this study revealed both positive and negative factors which appear to influence care of burn patients. The positive factors (motivation) need to be maintained, and immediate actions should be taken to address the negative (hindering) factors. Large scale studies to quantify these results are deemed necessary, and public health measures are needed to prevent burn injuries in children.

## Background

The World Health Organization (WHO) estimates that every year 265,000 deaths are caused by burns, and the majority of these deaths occur in low and middle-income countries [1]. It is estimated that 90% of the burn injuries which occur in low and middle-income countries is due to limited public health infrastructure [2]. Burn injury is recognized as the most devastating of all injuries and is considered a public health crisis [3, 4]. Furthermore, it is reported that burns are the 11th leading cause of death in children aged 1–9 years, and children under five years in the WHO African Region. This region has almost three times the incidence of burn deaths in infants compared to global averages [1]. In low-income countries, most burns take place in domestic settings [3, 5]. Most burn injuries are scaled burn resulting from hot liquids, electrical, chemical and intentional injury [6]. The extent of the injury depends on the degree of heat and length of time in contact with the heat [7]. Children have a relatively thinner dermis than adults, therefore for any given thermal insult, children typically sustain a deeper burn in comparison to adults [8].

Caring for patients with burn injury may be more problematic in low and middle-income countries due to inadequate resources. Many children suffering burn injuries may be at risk of severe complications if immediate quality nursing and medical care is not provided. Complications such as infections, decreased tissue perfusion, acute renal failure, contracture, and death have been reported [9]. Good quality management of burn injuries is crucial to relieve pain, prevent disfigurement, amputation of affected limbs, or even death, which may occur in severe cases. The management of pediatric burns and their sequelae remain demanding and extremely costly even in well-equipped, modern burn units of high-income countries [10]. A brief global review showed that first aid management of burns was received by 8.7%, and only 12.1% of cases applied cold water, which contributes to reduction in re-epithelialization time in children [11]. In Tanzania, a mini-meta-analysis revealed that only 14.3% of the victims applied cold water and 17.5% applied nothing, [12] implying knowledge deficit about pre-hospital burn care. In most low-income countries, late presentation to health facilities, lack of well-equipped burn centers and trained medical personnel significantly contribute to increased morbidity and mortality [13]. Review of pediatric burn care in sub-Saharan African countries revealed that provision of burn care is mostly influenced by availability of resources, trained personnel, and accessibility of health care facilities [14]. In Kenya, long distances to health facilities, poor transport systems from rural to urban areas where specialized burn care is available, limited space and supplies, and inadequate staffing in burn centers severely affect burn management [15]. Thus, the outcome of burn injuries is greatly influenced by the quality of care that patients receive, resources available, and skills level of health care providers [7]. In low-income countries, infection contributes to increased mortality and morbidity among burn patients [16].

A study conducted in Mwanza, Tanzania [17] on pediatric injuries reveal that 32 of 150 (21.3%) patients who participated in the study sustained burn injury. In Muhimbili National Hospital (MNH), burn injuries are a common indication for pediatric surgical admission and contribute significantly to high morbidity and mortality. In 2011, of the 351 patients who were admitted to the pediatric burn unit, 57 patients died due to wound sepsis, anemia, or severity of injury (unpublished unit report). In such a context, it is essential to understand the situation of care provided to hospitalized patients with burn injury. Therefore, the purpose of this article is to describe factors influencing the provision of nursing care to the hospitalized pediatric patients with burn injuries at MNH, Dar es Salaam, Tanzania.

**Fig. 1:**
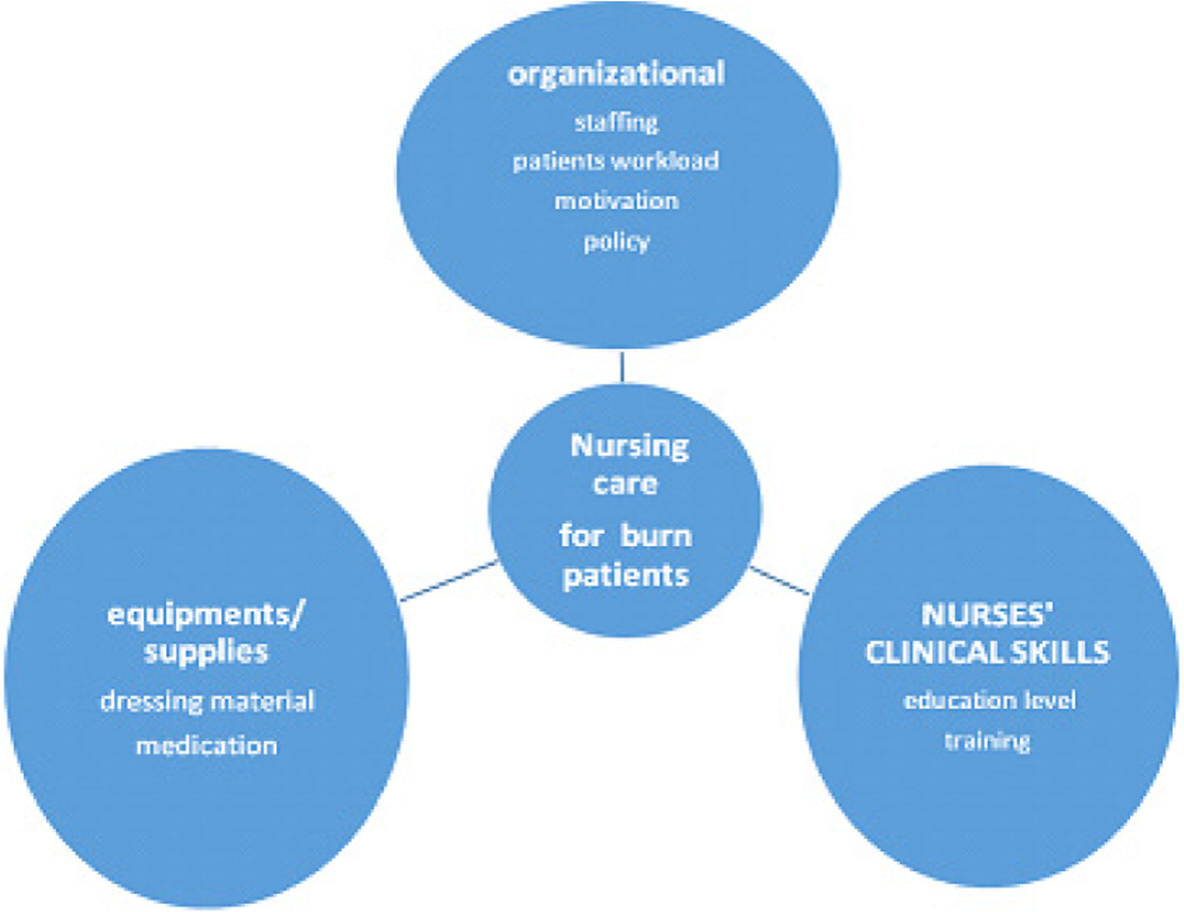
Modified conceptual model from concept analysis (Quirke et al. 2011)

A conceptual model by Quirke [18] has been modified and employed to explore factors influencing provision of nursing care to hospitalized pediatric burn patients. The original model was derived from a concept analysis of suboptimal care of the acutely ill ward patients in articles published between 1990 and 2009. This modified conceptual model consists of three components that contribute to nursing care to burn patients: organization of work (staffing, patients’ workload, motivation, and policy), availability of equipment/supplies (dressing material and medication) and clinical nursing competence (nurses’ clinical skills, education level, and training). The components of the model have been used to guide data collection, analysis, presentation and discussion of the findings (Fig. 1).

## Methods

### Design

A descriptive qualitative design was conducted to explore informants’ [nurses’] perceptions about factors influencing provision of nursing care to hospitalized pediatric burn patients. The modified conceptual model was used to guide the development of this research.

### Setting

This study was conducted in the pediatric burn unit at Muhimbili National Hospital (MNH). MNH is a university teaching hospital located in Ilala Municipal, Dar-es-Salaam, Tanzania. The hospital attends 1000 to 1200 patients per day. MNH consists of various departments. The department of surgery hosts two burn units; separated into adults and pediatrics. The pediatric burn unit has 26 beds. Often the number of children admitted exceeds unit capacity due to a large number of referral cases.

### Population

Nurses working in the pediatric unit were recruited into the study. During the study, the unit consisted of 18 full-time nurses. Of these, one was a bachelor degree holder, ten were diploma holders, and seven were certificate holders. During time of data collection, 16 nurses were available and two nurses were on annual leave. Of the 16 available nurses, 13 nurses met inclusion criteria. Therefore, 13 nurses were at MNH during data collection and had working experience of more than one year in a burn unit. The exclusion criteria included ill nurses and those who appeared to have no time for interview because of workload.

### Sampling procedure

Purposeful sampling was employed to recruit informants that could best inform the study [19]. Only nurses who had worked in the pediatric burn unit for more than a year and who agreed to participate in the study were recruited. Purposeful sampling ensures study participants have knowledge of the subject being studied. The potential informants were recruited through the nurse in charge according to their working shift.

### Data collection

Ten informants consented to take part after understanding the details of the study, which included their rights as informants. However, during data collection only five nurses were available for interview. Five informants were not available for interview because of overwhelming responsibilities of caring for seriously ill patients, and thus had no time to participate in interviews. The consented available informants were asked for the possibility of audio recording the interviews, and they agreed to be audio recorded. The audio recorder was used to ensure that the entire conversation was captured. The author used the interview guide to conduct the interviews, and sometimes was probing to get more information and clarification. The critical questions focused on: organization of provision of care, availability of equipment concerning the provision of care and nurses’ clinical skills on caring for burn patients. One interview session was performed with each informant. The interviews lasted between 25 and 45 min. Also, a simple observation was used to validate information provided in face to face interviews. The first author visited the units after interviews and took notes to ensure the integrity of collected data. The observation notes facilitated understanding of how nurses were providing care and resources used. Data collection took place in May, 2012.

### Data analysis

Data analysis was conducted after each interview. The analysis was an active, interactive, and continuous process. The audio recorded information was transcribed word for word. After transcribing the interviews, both authors read all transcripts several times to ensure no errors occurred during transcription. The first author translated the conversation from Swahili to English. Content analysis approach [20] was loosely employed in which transcribed texts were broken into meaning units. The two authors coded each transcript independently. Various codes were compared for similarities and differences and sorted into sub-categories and categories were formed. The authors discussed the analysis process and compared the categories for final consensus. Direct quotations from informants were added to ensure that informants’ concerns were reflected in the article. To protect informants’ identity, we did not include personal identifiers in the quotes. In this context, informants’ voices in supporting the presented views were more important than use of personal identifiers in the quotes.

### Reflexivity

Reflexivity was taken into consideration by ensuring that the author(s)’ prior experiences as health care providers or professional nurses did not influence the informants’ responses. Reflexivity was done through a prior introduction that the author was doing research rather than a nurse who might know the field.

## Results

### Socio-demographic characteristics

The informants consisted of five registered nurses who were working in the pediatric burn unit. All informants had been working in the burn unit between one and nine years. All informants were females. One nurse had special short training on burn care in pediatrics, and others were working through experience.

### Categories and sub-categories

The findings were divided into two major categories; motivating factors and barriers in the provision of burn care to pediatrics. The categories consisted of sub-categories as shown in Table 1.

**Table 1:** Categories and sub-categories

Sub-category	Category
Coordinated care of patients with burn (skills and team work) Involvement of family caregivers Availability of equipment Nurses’ commitment Job satisfaction	Motivating factors in provision of burn care
Inadequate staffing Patients’ overload Lack of standard skills Limited resources Cultural and socio-economic influence	Barriers in provision of burn care.

### Motivating factors in provision of burn care

#### Coordinated care of patients with burn (skills and team work)

The informants explained how proper skills facilitated provision of optimal care to pediatric burn patients. They explained that before establishment of the pediatric burn care unit in 2004, all burn patients were managed in the surgical ward with other surgical cases. The informants stated that having the pediatric burn unit in a separate place decreased the number of deaths and rate of infection in this population. Also, after the establishment of the burn unit, burn wounds were no longer disturbed by soaking in large dishes of water and Detrol solution. That method called an “open method,” was abandoned because it delays wound healing. The nurses reported that after implementation of the “closed method,” burn wounds were healing faster, the number of contractures decreased, and the infection rate among burn patients dramatically decreased. One informant commented about the shortfalls of the old method and praised the new one:

*“For anybody who sustains burn injury, if you leave the wound open, cold will pass through the flesh, and it [wound] dries up. Using this [old] method, once you want to perform dressing, you must remove the dried flesh by soaking, but currently we are not doing that. As a result, cases of contracture have decreased.”*

The informants said that teamwork facilitated provision of care to burn patients. They noted that burn patients needed multidisciplinary care to avoid complications associated with burn injuries. As such, the team consisted of a plastic surgeon, a nurse, a social worker, a nutritionist, and a physiotherapist. They said during major ward rounds, this multidisciplinary team reviewed and discussed patient care and management. This arrangement helped the whole team to understand patients’ needs better and contributed to patients’ recovery. For instance, they said when the dressing antibiotics were out of stock, relatives were asked to purchase these items. However, because of the teamwork, the social worker found donors that donated several tubes of Silverex cream for the patients.

#### Involvement of family caregivers

Informants observed that involving family caregivers in patient care facilitated recovery. They noted that family caregivers were the ones who spent more time with patients and needed to be aware of their responsibility towards patient outcomes. To facilitate this, informants said they provided health education in every morning shift. They said they provided training on hygienic behaviors, nutrition, prevention of burn injury, and complications associated with burn injury.

#### Availability of equipment

All informants appreciated hospital management for giving priority to the burn unit. The priority was done by supplying dressing materials such as gauze, Povidone iodine, and bandages. One informant stated that availability of working equipment made the environment conducive for providing care to the patients. Another informant emphasized that most of the dressing materials were consistently available in the unit. As such, once they were finished the attendants in the storeroom were informed, and the supply officer requested that the purchasing officer restock supplies accordingly. Another informant emphasized the availability of dressing equipment by saying that most of the time they received enough material unless there was a large number of patients:

*“Most of the time in the burn unit, we do not miss [supplies]; we are always given a priority.”*

However, some of the informants said sometimes they had to get equipment somewhere else because of limited supply within the hospital. One informant said they obtained support from neighboring hospitals to make sure all wounds of the patients which were supposed to be dressed on that day were dressed.

#### Nurses’ commitment

The informants said that their compassion, perseverance, gratification and sense of harmony in the pediatric burn unit enabled them to provide proper nursing care to burn patients. They believed that nurses in the burn unit had created a favorable environment in such a way that every new nurse who came in adapted to the situation. As a result, they believed that patient outcomes were improving. One informant described the situation:

*“The way we feel for these children, it hurts us a lot. Sometimes we take them as our children, and we feel sorry for their suffering. Sometimes we perform dressing until time for changing shift; we feel sorry to leave other children unattended. So we continue until we finish dressing all of them.”*

Other informant expressed their feelings about perseverance in caring for burn patients despite the overwhelming job:

“*Caring for a burnt patient is a very tough job, [she shook her head as a sign of sadness] … As you have seen when you start dressing procedures, it is not possible to stop and leave to look for food. So, you find most of the time we stay without eating until we go back home [low tone of voice].”*

They emphasized that in order to be successful in their daily activities, they often invited God. They stated that within the unit, a routine of praying together in the morning was done prior to beginning patient care. They realized that this habit increased harmony among them to achieve their daily objectives peacefully.

#### Job satisfaction

Informants perceived their responsibility of caring for burn patients as a rewarding and gratifying experience. They felt that if someone is providing care and another person appreciates what has been done, it increases providers’ job satisfaction. They said this was observed in the pediatric burn unit, where some patients’ relatives came back to show their gratitude. They noted that this habit of appreciation from care recipients increased their performance. One informant described this:

“*Most of the time we receive information from the director of hospital that there were people who came into her office to appreciate the good care which they got for their children. Apart from that some relatives came directly to the ward for thankfulness [she smiled]. I think we are doing good job.”*

Further, the informants believed that patients with burn are managed better at MNH than in other hospitals in Dar-es-Salaam. This was evidenced by patients who were resistant to go to peripheral hospitals after being discharged from the burn unit. They said patients requested to continue to be cared for at MNH instead of continuing with care in the peripheral hospitals. Moreover, the nurses felt satisfied after taking care of severely burnt patients who recovered fully. They appreciated all efforts they undertook to enhance patient survival, and they said such feelings of satisfaction brought happiness in their work. Below is an example:

*“There were five patients from Railway station who sustained burn injury almost all over the body, but it took only one week to recover.”*

### Barriers in caring for burn patients

Although the informants demonstrated various aspects of motivation in caring for burn patients, sometimes they encountered problems. These included inadequate resources such as human (staffing), and non human resources (lack of water, sterilized materials, dressing, drugs), lack of standard skills, and poor socio-economic influence.

#### Inadequate staffing

The informants mentioned that scarcity of human resources, especially nurses, affected provision of care to burn patients. Even though this problem was at an organizational level, more effect was experienced in the burn unit than in other units because of increased needs among pediatric burn patients. Burn injury patients needed routine monitoring regarding feeding, fluids intake and output, wound dressing and general cleanliness. The nurses working in the burn unit were the ones to supervise all these needs. Due to a shortage of nurses, it was difficult to manage all these activities properly. As a result, some patients suffered from malnutrition and septicemia.

This staffing shortage increased the workload among the few available nurses. As a result they became tired and failed to do extra activities. Some of them expressed that they had fallen behind on patient care due to exhaustion. One informant reported:

*“You see, here I have decided to leave other activities and start to enter specimens into the computer; some people want syringe for feeding; I don’t know what! You see up to now, we have not started to dress wounds!”*

They said burn care was a very exhausting task that requires dedicated and committed health care workers. Nurses in the burn unit experienced physical and emotional exhaustion in the provision of care. Due to an inadequate number of nurses, the available nurses sometimes had to stand for six hours while performing dressing changes. One informant described how the situation affected them:

*“I have only one year since I started working in this unit, but I have already developed varicose veins.”*

Due to the heavy workload, informants said that there was no motivation to increase their working morality. They expressed feeling a lack of motivation by stating that burn care is a difficult task which requires sufficient energy and good health. They complained that standing from 9 AM to 3 PM performing dressing changes was not easy to sustain.

#### Patient overload

The informants noted that the incidence of burn injuries in the pediatrics burn unit was increasing over time. Some informants compared the available space and the number of admitted patients. One informant reported that she noted an increased rate of admission within one year of her experience in the burn unit. She said when she started working in the burn unit, there were 14 or 15 patients in the ward. This number had doubled, to the point in which two patients were sleeping in one bed, and some of them were admitted to the surgical ward. She concluded that there was a tremendous increase in cases of burn injuries.

Another informant added that many patients with burn injuries were referred to MNH, resulting in congestion within the burn unit. The unit only has two rooms with 26 beds, and these beds were very close to each other. She experienced discomfort when providing care because all patients, regardless of wound status [clean and septic], were admitted to the same room. She emphasized:

*“I think we need adequate space in order to separate patients [clean and septic cases]. For example, new admissions could have own room. Once they gain good progress, they could be shifted into another room, and if patient develops malnutrition or diarrhea, he/she should be shifted into a separate room. Also, patients who develop septicemia should be separated from patients with a clean wound.”*

#### Lack of standard skills

The informants expressed that no strict criteria existed for selecting staff to work within the burn unit. They stated that any registered nurse could be allocated to work in the burn unit. As a result*,* they developed a method of teaching one another in order to facilitate consistency in the provision of care to burn patients. The more experienced nurses were responsible for sharing experiences and training new nurses and students who were rotating within the unit. Thus, the nurses were providing a supervisory role to students in addition to their many other, time-consuming tasks.

Although informants said they shared knowledge each other, they lacked standard burn care skills because they were providing care on a routine basis. One informant saw this routine work as a problem and expressed that they needed more knowledge and skills in caring for burn patients. She said if there is education on burn care available, they would need to be considered in order to advance their knowledge and skills.

#### Limited resources

Both human and non-human resources were inadequate to meet the needs of patients who sustained burn injury. Despite prioritizing dressing material, important resources like laboratory equipment for collecting specimens was missing. Burn care needed a multidisciplinary approach, and a laboratory was the critical area where diagnosis of the underlying problem like causes of fever takes place. Due to a scarcity of laboratory equipment, it was challenging to manage fever properly. One informant explained how this problem affected the provision of care. She stated:

*“For burn patients, we need to take pus swab several times, but you find no equipment … You find patients with running fever but no equipment for pus swab.”*

Also during data collection, informants reported a shortage of supplies for wound swab and blood culture bottles. So, when patients were suspected to be infected, they were given antibiotics without performing drug sensitivity tests beforehand. They said this could interfere with the confirmation of patients’ diagnosis. As a result, patients could be suffering from fever and other complications like anemia and malnutrition without proper diagnosis. Also dressing antibiotics, such as Silverex, was inconsistently available.

Although in the sterilization center, material such as gauze was sterilized, health care providers still came across discrepancies which hindered the provision of optimal care. Some informants said that in the tray center they found only one towel while they needed two: one to put on the mackintosh and another one for covering patients. Instead, they placed patients on mackintosh directly while that area is frigid for the patients. Furthermore, informants noted that there was no machine for linen sterilization in the unit:

*“The unit could have a special sterilization machine for cleaning linen because when we collect pus swab for culture and sensitivity, the bacteria found, are similar to those found in the hospital environment.”*

Lack of water for hand washing was a significant concern in the pediatric burn unit. All informants complained about this as it hindered the provision of proper care. They stated that water is the mainstay of infection prevention control. One informant stated:

*“In the unit there is no water; nurse attendants were supposed to go to fetch water from other places to wash hands before and after dressing procedures.”*

Another informant added that due to inadequate running water in burn unit, there were frequent outbreaks of diarrheal diseases among patients.

#### Cultural and socio-economic influence

The informants noted that family culture and socio-economic status had a notable impact on wound healing. They noted that poor patients’ socio-economic status and culture often resulted in poor nutritional status and delay in seeking care at the hospital. Furthermore, they said most of the patients who were admitted to the burn unit were less than five years old. These children were at high risk of infection and burn injury added more risk to the group. Therefore, if patients had malnutrition or diarrhea, the healing process was slowed. One informant said:

*“Lack of income results into poor living condition, in turn, causes lack of required nutrients for the patients. Poor patient nutritional status results in longer patient stays in the ward. Therefore, as a nurse, I fail to achieve good outcome meaning patient recovery time and return home”*

Furthermore, the informants noted that patients with low socio-economic status delayed coming to the hospital and they were the ones who presented with septic wounds. Thus, it became difficult for such patients to heal and those are the ones who would die of wound sepsis. Also, they said once a patient is admitted in the ward with high total body surface area of burn like 60%, it is difficult to manage such a patient. One informant stated:

“*One day we received a young boy of about seven years old who sustained burn injury with high percentage. We stayed with him for about 15 hours and then he died because the burn injury extended to the brain. I observed some discharge through ears, [the nurse lowered her voice and clenched her face] that means he was excessively burnt to the brain.”*

Overall, both motivating factors and barriers were crucial to influencing the provision of optimal care to the pediatric patient with burn injuries.

## Discussion

Provision of care for burn injury patients is positively influenced by coordination of staff regarding skills and teamwork, involvement of family caregivers, availability of most equipment, nurses’ commitment and job satisfaction. On the contrary, inadequate staffing, patient overload, lack of standard skills, limited resources and poor socio-economic status among families appear to hinder optimal care to burn injury patients. In discussing the key findings, we loosely drew upon selected components of the modified conceptual model by Quirke [18] as a guide. According to this model, optimal care of patients with burn injuries requires coordination of staffing, nurses’ clinical skills, and availability of equipment. These components may contribute to optimal care and thus alleviate adverse outcomes among patients with burn injury.

In the present study, it is worth noting that shortage of staff affects the provision of optimal care. It appears that the few available nurses were overwhelmed by the workload imposed on them. Subsequently, this made it difficult to provide quality nursing care. Previous studies [21, 22] showed that a heavy nursing workload was associated with suboptimal patient care. Furthermore, heavy work load negatively affects nursing job satisfaction and, as a result contributed to high job termination and the nursing shortage leading to suboptimal care of patients [23]. Also, another study documented that staffing affected patient outcomes, increased medical errors, increased patients’ length of hospital stay, and increased patient mortality [24]. However, in the present findings, teamwork helped to meet patients’ needs in the sense that the composition of a multidisciplinary team approach was highly successful in achieving patients’ holistic management and care. Thus, positive outcome amongst patients with burn injuries depends on teamwork and efficient collaboration among the team (i.e., nursing, physicians, physiotherapists, and nutritionists).

The training among nurses appears to best influence the provision of optimal nursing care. For instance, when it comes to nurses’ clinical skills, the use of “closed wound” method is worth noting. Use of this new knowledge led to abandonment of the “open wound” method, which disturbed the wound by soaking in water. The use of “open wound” in turn, affected the healing process. In this case, a proper assessment technique seems to be necessary because it led to sound decision-making in wound care. A study in England documented that poor assessment skills in nursing and medical staff and lack of recognition of patients’ deterioration were the attributes of suboptimal care [25]. While recognition and interpretation of physiological abnormalities is primarily a nursing responsibility, it is high time to sensitize nurses to prioritize physiological needs of critically ill patients. The ability to recognize physiological abnormalities is a crucial factor in the prevention of an impending adverse event. A previous study showed that suboptimal care implies lack of knowledge related to management of airway function, circulation, and functions of other systems [26]. Complications that arise after burn injury require burn nurses to have a broad-based knowledge of standard burn care. This includes wound care techniques, critical care techniques, diagnostic studies, rehabilitative, and psychosocial skills. Thus, lack of implementation of standard care may affect the provision of optimal and holistic patient care.

Furthermore, the availability of essential equipment in burn units is crucial. Burn units must have access to the necessary equipment to facilitate the provision of quality care. Such equipment includes a vital signs machine with cardiac monitoring capabilities, equipment for laboratory tests such as wound culture swabs, and blood culture bottles. This equipment will aid in improved patient care and the ability to rule out infection. Also, the availability of adequate consumables such as gauze, cotton wool, syringes, and intravenous cannulas will aid in facilitating proper patient care. In most cases, burn patients may be at risk of getting infections if sterile sheets do not cover them. Thus, good performance of nurses is enabled by a supportive working environment, which includes having adequate equipment and supplies. Furthermore, a supportive working environment includes continual improvement of organizational issues, such as decision-making and information-exchange process issues, and capacity issues such as support services and infrastructure. The availability, reliability and consistent supply of equipment between clinical areas have been noted to contribute to the quality of care to patients [27]. However, difficulties in obtaining or being unfamiliar with equipment due to the number and variety of devices in hospitals have been reported to cause suboptimal care to the patients [28]. This evidence shows that nurses should have a formal initial training upon hire, and ongoing professional training. This training will aid nurses to incorporate knowledgeable, relevant, and up to date skills into their patient care, thus ensuring positive patient outcomes. In South Africa, burn care varies in terms of the organization, clinical management, facilities, staffing, workload and outcome [29]. A mini-meta-analysis from Tanzania showed that lack of correct and useful knowledge about immediate action after injury hindered initial treatment of a burn amongst caregivers [12]. Consequently, the lack of standardized burn care protocols in hospital settings, and the lack of community education on initial burn care have a significant impact on burn care and patient outcomes in Africa.

### Limitations

The findings cannot be generalized to all Tanzanian nurses in pediatric burn units, as the present study recruited only five nurses. Also, more data could have been obtained if a series of interviews had been conducted with each nurse. However, the available findings add value concerning the challenges and success of burn care from the perspectives of nurses. Furthermore, information in this paper may be useful in the future care of burn patients in Tanzania and elsewhere.

## Conclusions

This small study in a specific local context highlights various factors that influence the provision of care of pediatric patients with burn injuries. The motivating factors such as teamwork, availability of equipment, nurses’ commitment and job satisfaction should be maintained to improve patient outcomes. On the other hand, the barriers to achieving optimal care of patients with burn injuries such as shortage of staff, patient overload, lack of standard skills among nurses, socio-economic influence, and lack of resources may greatly affect the provision of optimal care, and thus should be addressed. Overall, the findings emphasize the important efforts made by health care workers particularly nurses, in meeting patients’ needs despite limited resources.

### Implications

#### Practice

Evidence shows that nurses are the key team members to ensure the care of pediatric burn injuries are of good quality and comprehensive. They are in a better position than other team members to note critical changes that require immediate attention, prevention of infections, and adequate pain management.

#### Education

The current study contributes to the evidence on factors influencing the provision of care to burn injuries in low-income settings. Hospital management and nursing institutions can use these findings to improve burn care in similar contexts. This evidence highlights the need for nurses to educate about burn prevention and proper burn care both in the hospital and the community settings.

#### Policy

Policymakers may use these findings to enhance public measures for burn prevention education in their communities, and to advocate for the availability of proper equipment and medicines in burn units in their local hospitals.

#### Research

More studies may be carried out in the area of burn care, particularly among children in low socio-economic groups. Also, longitudinal quantitative studies may shed more light on factors affecting the provision of care to hospitalized pediatric patients with burn injuries.

## Abbreviations

MNH: Muhimbili National Hospital
MoHCDGEC: Ministry of Health Community Development Gender Elderly and Children
WHO: World Health Organization
